# Fed-Batch Cultivations of *Rhodospirillum rubrum* Under Multiple Nutrient-Limited Growth Conditions on Syngas as a Novel Option to Produce Poly(3-Hydroxybutyrate) (PHB)

**DOI:** 10.3389/fbioe.2019.00059

**Published:** 2019-04-02

**Authors:** Stephanie Karmann, Sven Panke, Manfred Zinn

**Affiliations:** ^1^Institute of Life Technologies, University of Applied Sciences and Arts Western Switzerland (HES-SO Valais-Wallis), Sion, Switzerland; ^2^Department of Biosystems Science and Engineering, ETH Zurich, Basel, Switzerland

**Keywords:** *Rhodospirillum rubrum*, syngas, fed-batch, poly(3-hydroxybutyrate) (PHB), multiple nutrient-limitation, gas transfer, maintenance energy, redox potential

## Abstract

Syngas from gasified organic waste materials is a promising feedstock for the biotechnological synthesis of the bioplastic poly([*R*]-3-hydroxybutyrate) (PHB) with *Rhodospirillum rubrum*. In a first approach, growth studies were carried out with this strain in gas-tight serum vials. When syngas (40% CO, 40% H_2_, 10% CO_2_, and 10% N_2_ v/v) was diluted with N_2_ to 60%, a 4-fold higher biomass production was detected compared to samples grown on 100% syngas, thus indicating a growth inhibitory effect. The best performing syngas-mixture was then used for C-, C,N-, and C,P-limited fed-batch fermentations in a bioreactor with continuous syngas and acetate supply. It was found that C,P-limited PHB productivity was 5 times higher than for only C-limited growth and reached a maximal PHB content of 30% w/w. Surprisingly, growth and PHB production stopped when N, as a second nutrient, became growth-limiting. Finally, it was concluded that a minimal supply of 0.2 g CO g^−1^ biomass h^−1^ has to be guaranteed in order to cover the cellular maintenance energy.

## Introduction

In 2014 almost half of all industrial and municipal waste produced in the European Union (EU) ended up in landfills (Eurostat, 2014). This implies a negative impact on the surrounding soil and groundwater by leachates (Kjeldsen et al., 2002) and a loss of carbon that could be reused as a “third generation” feedstock for biotechnological products. The organic waste fraction, from agriculture, wastewater treatment plants, food waste, or some other municipal wastes, can be recycled by microorganisms through anaerobic digestion, forming valuable products like biogas and fertilizer (Holm-Nielsen et al., 2009; Mao et al., 2015). However, municipal wastes are highly complex and not all types of carbon, such as lignin and hemicellulose from agricultural wastes, are easily accessible to microbial fermentation.

An alternative to direct fermentation is the use of syngas, the product of pyrolyzed and gasified organic matter (Demirbas, 2002; Beneroso et al., 2014). Syngas is rich in CO, H_2_, and CO_2_ and can contain traces of other small hydrocarbons such as methane, ethane or ethylene. Syngas is also an abundant waste product from industrial processes including steel milling or petroleum refining (Köpke et al., 2011), making it an important potential feedstock for further conversion. In fact, the chemical conversion of syngas to fuel-ethanol, butanol, acetic or butyric acid at high temperatures and elevated pressure has been applied for many years (Ail and Dasappa, 2016). Autotrophic microorganisms can also transform CO from syngas into value-added products including bioethanol, acetic acid, 2-butanol or *n*-propanol at more moderate environmental conditions such as ambient pressure and temperature (Henstra et al., 2007; Munasinghe and Khanal, 2010; Köpke et al., 2011; Bengelsdorf et al., 2013; Liew et al., 2013).

Poly(3-hydroxyalkanoates) (PHAs) are interesting products that can be synthesized by syngas fermenting bacteria (Do et al., 2007). These biodegradable polyesters offer an alternative to petrochemical plastics (Sudesh et al., 2000). Although there are already numerous applications for PHAs in the field of biomedicine or food packaging (Chen and Wang, 2013), broader competition with petrochemical plastics is limited by price and availability. Economic analyses showed that the carbon source, e.g., glucose, contributes almost 30% to the final cost of PHA produced by engineered *Escherichia coli* (Choi and Lee, 1997). Hence, low cost carbon sources such as syngas could significantly lower the final price. Finally, syngas neither competes with the food chain nor does it require agricultural land and water for its production.

**Figure 1:**
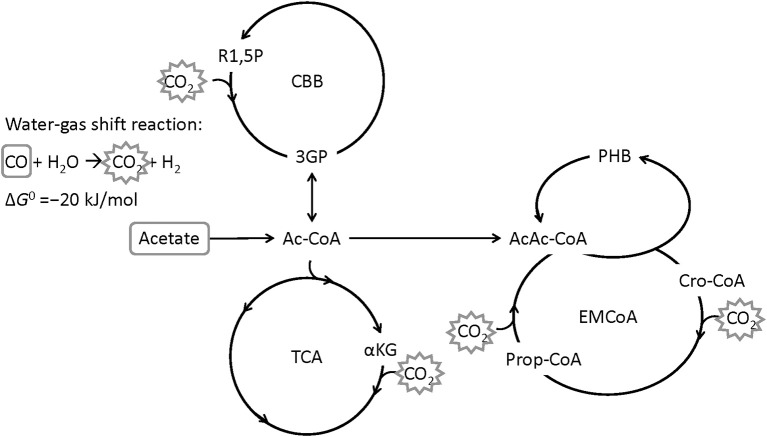
Simplified metabolic map of *R. rubrum* with CO and acetate as carbon source. The water-gas shift reaction is converting CO to CO_2_ by gaining energy (Najafpour et al., 2004). CO_2_ can then be fixed through the Calvin-Benson-Bassham cycle (CBB), the tricarboxylic acid cycle (TCA), or the ethylmalonyl-coenzyme A cycle (EM-CoA). R1,5P: ribulose-1,5-bisphosphate, 3PG: 3-phosphoglycerate, Ac-CoA: acetyl coenzyme A, αKG: α-ketoglutarate, AcAc-CoA: acetoacetyl-CoA, Cro-CoA: crotonyl-CoA, PHB: poly([*R*]-3-hydroxybutyrate) (Modified from Revelles et al., 2016).

The bacterium *Rhodospirillum rubrum* is able to use CO from syngas as carbon and energy source for growth and for the production of the PHA poly(3-hydroxybutyrate) (PHB) under anaerobic conditions (Do et al., 2007). *R. rubrum* is a Gram-negative, facultative photosynthetic purple non-sulfur bacterium which is able to grow hetero- or autotrophically, aerobically or anaerobically (Schultz and Weaver, 1982; Najafpour and Younesi, 2007; Rudolf and Grammel, 2012; Narancic et al., 2016). *R. rubrum* serves as a model organism for studies on nitrogen fixation (Lehman and Roberts, 1991), hydrogen production (Younesi et al., 2008) or the production of photosynthetic membranes (Grammel et al., 2003). CO-assimilation for growth and PHB production is assumed to proceed via the enzyme CO dehydrogenase (EC 1.2.7.4, CODH) that catalyzes the water-gas shift reaction, during which CO is oxidized with H_2_O to CO_2_ and H_2_ (Kerby et al., 1992, 1995). CO_2_ can then be assimilated by the ethylmalonyl-CoA pathway, the Calvin-Benson-Bassham cycle or the reductive tricarboxylic acid cycle (Revelles et al., 2016). Acetate has been shown to be a good co-substrate for growth of *R. rubrum* on syngas, in particular with a positive effect on PHB accumulation (Revelles et al., 2016; Karmann et al., 2017). Figure 1 shows a simplified metabolic route of CO and acetate in *R. rubrum*.

Typically, PHA accumulation in bacteria is favored by unbalanced growth conditions providing an excess of carbon under a limitation by nitrogen (N), phosphorus (P), oxygen (O), potassium (K), or other essential nutrients (Kim and Lenz, 2001). One of the highest PHB quantities obtained so far, namely 232 g L^−1^ with a cellular content of 82% w/w was produced by the wild-type strain *Cupriavidus necator* grown in a P-limited fed-batch fermentation on glucose (Ryu et al., 1997). In *R. rubrum*, PHB contents of 20 or 46% w/w were reached under anaerobic, photosynthetic, N-limiting conditions, and 30 mM acetate or 30 mM β-hydroxybutyrate as sole carbon source, respectively (Brandl et al., 1989). In syngas shake flasks supplemented with 10 mM sodium acetate, a PHB content of 28% w/w was found at a low cell density of an optical density (OD_600_) of 1 (Revelles et al., 2016).

To achieve the goal of economical PHB production with *R. rubrum* on syngas, the production process needs to be better understood and optimized. Therefore, the objective of this study was to identify the growth conditions under which the PHB production from syngas by *R. rubrum* is optimal. First, we explored PHB production in serum vials with different syngas compositions and second in fed-batch fermentations with controlled acetate feeding under nutrient-limiting regimes including C (CO and acetate), dual C,N and dual C,P limitation. With this continuous feeding strategy, we could establish multiple nutrient-limited growth conditions, which have been shown to be maximal for PHA production for *Pseudomonads* among others (Durner et al., 2000; Wisuthiphaet and Napathorn, 2016). Indeed, best performance was found for simultaneous carbon (CO and acetate) and phosphorus limitation leading to biomass containing 30% w/w PHB.

## Materials and Methods

### Bacterial Strain, Growth Conditions, and Media

*R. rubrum* S1 (ATCC 11170), stored in aliquots at −80°C in 16% v/v glycerol, was used for all fermentations. Liquid cultures were grown in modified RRNCO medium (Kerby et al., 1995) as described previously (Karmann et al., 2017).

Precultures were grown in flasks as follows: Sterile 1,000 mL bottles (Müller + Krempel, Bülach, Switzerland) closed with a butyl rubber septum and a screw cap or 100 mL serum vials closed with a 1 cm butyl rubber stopper and an aluminum crimp cap were filled to 50% v/v with aerobic modified RRNCO medium supplemented with 2.7 g L^−1^ (15 mM) fructose (pH 7.0) through a sterile 0.22 μm filter. The preculture was inoculated with a sterile syringe and needle directly from a cryo-vial (OD_600_ = 3.33) resulting in an initial optical density of OD_600_ = 0.08 and a glycerol concentration of 0.38% v/v.

Incubation followed in an orbital shaker at 30°C and 180 rpm for 48 h. During this time no gas exchange between the culture headspace and the surrounding air was possible resulting in a continuous decrease in pO_2_ and a slow adaptation to anaerobic growth conditions.

### Batch Experiments on Syngas in Serum Vials

Initial tests to assess growth on syngas were performed in closed 100 mL serum vials filled with 20% v/v sterile-filtered modified RRNCO medium containing 0.82 g L^−1^ sodium acetate (10 mM or 0.59 g L^−1^ acetate). Air had to be removed from the headspace prior to filling with syngas. Therefore, each capped serum vial was exposed twice to 1 min vacuum followed by re-filling with N_2_. After a third vacuum cycle the headspace of the serum vial was filled with a commercially available syngas-mixture containing 40% CO, 40% H_2_, 10% CO_2_, and 10% N_2_ v/v (Pan Gas, Dagmersellen, Switzerland), which was in some cases diluted with N_2_ as indicated later. A sample containing only N_2_ in the headspace was used as negative control. Right before inoculation, 1 mL of autoclaved, anaerobic 1 g L^−1^ Na_2_S·9H_2_O reducing agent stock solution was added per L of medium.

For each growth condition, triplicates were inoculated from the same preculture (final OD_600_ = 2.5) with 0.64 mL to reach a starting OD_600_ of 0.08. The vials were incubated horizontally in the dark at 30°C and 180 rpm. Every 24 h vacuum was applied again to exchange the headspace gas with fresh syngas though a sterile needle and filter. Culture samples were taken every 24 h with a sterile needle and syringe to determine the OD_600_.

### Bioreactor Settings and Fed-Batch Growth on Syngas

The syngas fermentation platform and the fermentation settings have been described in detail earlier (Karmann et al., 2017). All fed-batch fermentations were carried out in a 3.6 L bioreactor (Labfors 5, Infors AG, Bottmingen, Switzerland) with an initial working volume of 2 L of modified RRNCO medium containing 0.59 g L^−1^ acetate. In case of the N-limited fed-batch the NHCl concentration was reduced from 1 g L^−1^ to 383 mg L^−1^ (equals 100 mg L^−1^ NH_4_-N), in case of the P-limited fed-batch, the phosphate buffer (KH_2_PO_4_ and K_2_HPO_4_) was omitted.

The continuous feeding of acetic acid at a concentration of 120.1 g L^−1^ in water started typically after 43 h of cultivation, when most of the initial acetate was consumed. The feeding rates were adjusted two times per day, based on the culture OD_600_ as described previously (Karmann et al., 2017).

The redox potential was recorded continuously with an inline probe (Hamilton Bonaduz AG, Bonaduz, Switzerland). The off-gas composition was measured continuously with a quadrupole mass spectrometer (MS) (QIC Biostream, Hiden Analytical, Warrington, UK). Samples were taken during the bioprocesses for the quantification of biomass (cell dry weight (CDW) by gravimetry, OD_600_ by spectrophotometry and total cell count (TCC) by flow cytometry (FCM), dissolved CO (DCO) with a myoglobin assay, PHB content by FCM and gas chromatography (GC), acetate by high pressure liquid chromatography (HPLC), and ammonium nitrogen (${\text{NH}}_{4}^{+}$-N) by spectrophotometric test kit. These analytical methods were described previously (Karmann et al., 2016, 2017).

### Phosphorus (P) Quantification

Supernatant samples (3,900 g, 4°C, 10 min) were stored at −20°C until ICP analysis. To this end, a 10 mL aliquot of culture supernatant was thawed and acidified with 3 drops of 69% (v/v) HNO_3_ (TraceSELECT®, Fluka) and analyzed with a Varian 720-ES inductively coupled plasma—optical emission spectrometer (ICP-OES). Emissions were recorded at 213.6 and 214.9 nm. The measurements were validated with a calibration curve ranging from 0.01 to 50 mg L^−1^ P (H_3_PO_4_ + H_2_O, TraceCERT®, Sigma-Aldrich).

### Elemental Analysis

Bacteria were harvested by centrifugation (3,900 g, 4°C, 10 min) and the cell pellet was washed once with 0.9% aqueous NaCl. The pellet was stored at −80°C for at least 24 h and then freeze-dried at −80°C at a pressure of 0.25 mbar in a freeze dryer (Cryodos, Telstar, Terrassa, Spain). Approximately 1.3 mg of freeze-dried biomass was then digested by combustion. Combustion products were analyzed with a LECO TruSpec Micro. Carbon (as CO_2_) and hydrogen (as H_2_O) were quantified by infrared spectroscopy. The nitrogen content (as N_2_) was determined by a thermal conductivity detector. All samples were analyzed in duplicates.

### Calculations

Biomass yields (Y_X/S_) (Equation 1) on acetate, CO, N, and P (s) were based on the cell dry weight (x), and PHB yields were calculated using the PHB and substrate (s) concentrations, both in g L^−1^ according to Equation (2) The actual dilution rate (D) was calculated by the division of medium feed (F) by the actual working volume of the culture (V) (Equation 3). Specific biomass (q_x_; Equation 4) and PHB (q_PHB_; Equation 5) production rates were calculated and the volumetric biomass (P_x_; Equation 6) and the volumetric PHB (P_PHB_; Equation 7) productivities determined.

(1) $$\begin{array}{l} Y_{X/S}=\frac{\Delta x}{\Delta s} \end{array}$$

(2) $$\begin{array}{l} Y_{PHB/S}=\frac{\Delta PHB}{\Delta s} \end{array}$$

(3) $$\begin{array}{l} D=\frac{F}{V} \end{array}$$

(4) $$\begin{array}{l} q_{x}=\;Y_{X/S}*D \end{array}$$

(5) $$\begin{array}{l} q_{PHB}=Y_{PHB/S}*D \end{array}$$

(6) $$\begin{array}{l} P_{x}=x*D \end{array}$$

(7) $$\begin{array}{l} P_{PHB}=PHB*D \end{array}$$

## Results and Discussion

### Growth of *R. rubrum* in Serum Vials With Different Syngas Concentrations

**Figure 2:**
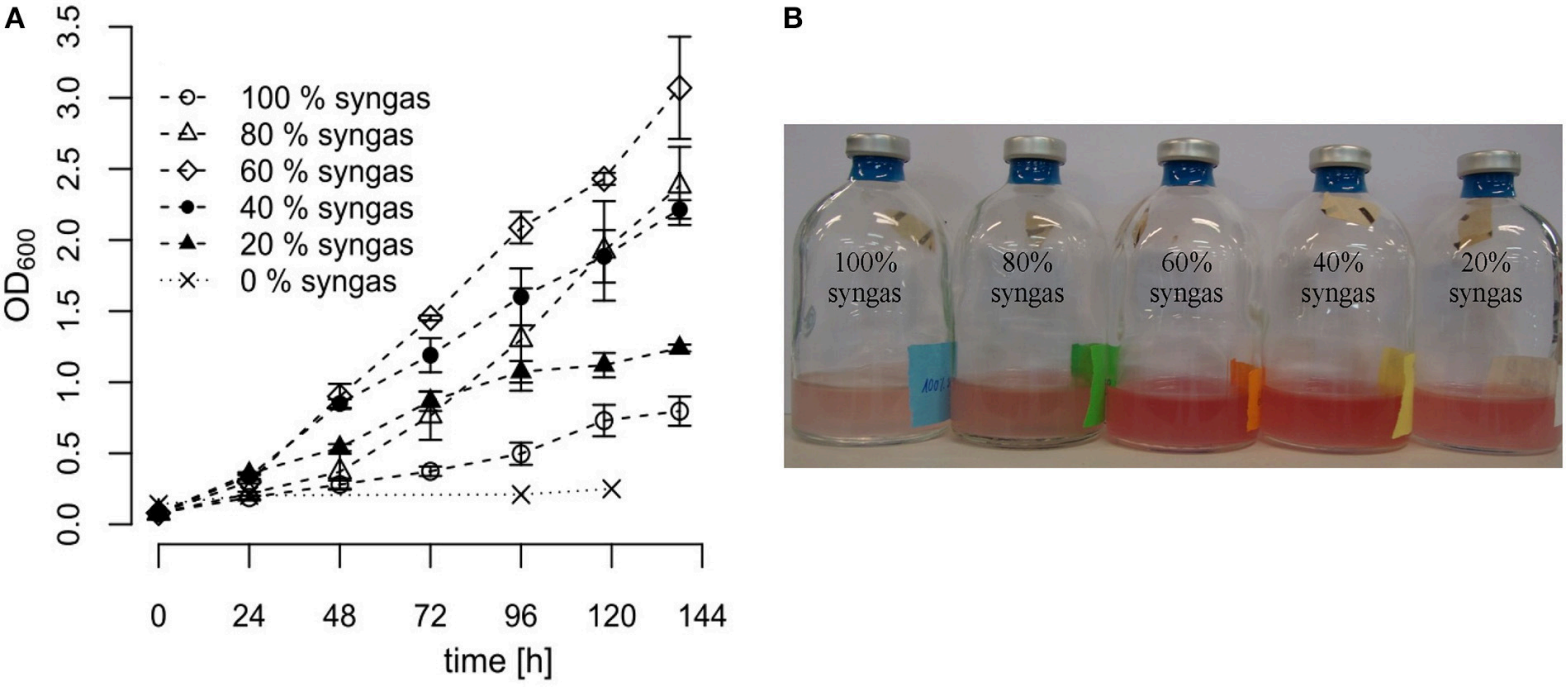
Growth of *R. rubrum* in serum vials with different syngas concentrations. *R. rubrum* cultures were grown on modified RRNCO medium containing 0.59 g L^−1^ acetate and the indicated syngas concentration in the headspace. The headspace filled with 100% syngas contained 40% CO, 40% H_2_, 10% CO_2_, and 10% N_2_ v/v. The other samples were diluted with N_2_ as indicated. **(A)** Growth curves are based on OD_600_ measurements. The error bars indicate the mean ± standard deviation determined from three independent replicates. **(B)** The *R. rubrum* culture in serum vials after 96 h of incubation. The 0% syngas control sample was colorless.

Different syngas mixtures were applied in order to investigate whether variation in the gas composition offers an opportunity to improve growth of *R. rubrum*. Therefore, we diluted the commercial syngas mixture (40% CO, 40% H_2_, 10% CO_2_, and 10% N_2_ v/v) with nitrogen to achieve different volumetric syngas contents ranging from 0% (only N_2_) to 100% (only commercial mix) and used it as the gas phase for growth experiments in serum vials. In all cases the liquid medium contained 0.59 g L^−1^ acetate as co-substrate. In general, OD_600_ measurements suggested that cultures grew linearly instead of exponentially, pointing toward CO-transfer from the gas to the liquid phase as a limiting step. As can be seen in Figure 2, differences in growth of *R. rubrum* as a function of the tested syngas mixture started to become detectable already 48 h after inoculation. After 138 h of incubation, the cultures with only 60% syngas in the headspace had grown to the highest OD_600_ of 3.07 ± 0.37, which was a 4 times higher biomass than for the samples with 100% syngas in the headspace (OD_600_ of 0.73 ± 0.1).

With the condition 100% syngas and the control sample containing only N_2_ we could reproduce the results from previously published experiments with *R. rubrum* using the same syngas composition and 0.59 g L^−1^ acetate as co-substrate (Revelles et al., 2016). Furthermore, from the control sample containing only N_2_ in the headspace, we concluded that acetate cannot be metabolized by *R. rubrum* under anaerobic conditions if there is no syngas available (Figure 2A). Finally, the results presented in this study show that different syngas concentrations do have a large impact on the growth of *R. rubrum* and offer therefore a target for process optimization. However, based on these shake-flask experiments, we were not able to conclude if the differences in growth occurred because of CO substrate toxicity or inhibition by the H_2_ or CO_2_ contents.

For *R. rubrum*, CO substrate toxicity has not been observed previously to the best of our knowledge. Previous experiments with a similar setup showed that *R. rubrum* doubling times inversely relate to the CO content in the gas phase (with 4.8, 5.7, and 8.4 h for 25, 50, and 100 CO%, respectively) but the sample grown on 100% CO reached the highest final biomass after 70 h of incubation (Kerby et al., 1995). Growth and PHB production as a function of CO content in the gas phase were investigated for *Pseudomonas carboxydohydrogena*, and there was no inhibitory effect of increasing CO concentration in syngas (from 10 to 30% v/v). The increasing CO content had a positive influence on the biomass production, but did not influence the cellular PHB content (Volova et al., 2015).

In general, the composition of syngas varies depending on its origin or substrate. Therefore, a syngas with the desired composition necessary to reach maximum growth rates can be tailored only to some extent. For example, pyrolysis of microalgal biomass was leading to CO contents that varied between 20 and 45% for different pyrolysis temperatures (Beneroso et al., 2013), whereas syngas as waste from the steel industry contains between 40 and 70% CO (Molitor et al., 2016). Consequently, the production of a syngas with the desired composition that favors *R. rubrum* growth will be a bioprocess-determining factor.

A study that systematically analyzes the effect of individual gas concentrations on growth of *R. rubrum* still remains to be done. However, since the advantages were considerable, syngas diluted to 60% was also used for the fed-batch fermentations in the bioreactor throughout this study. An additional, artificial contamination of this synthetic syngas with hydrocarbons, such as methane, ethane, or ethylene was not possible for technical reasons.

### Multiple Nutrient-Limited Fed-Batch Fermentations

**Figure 3:**
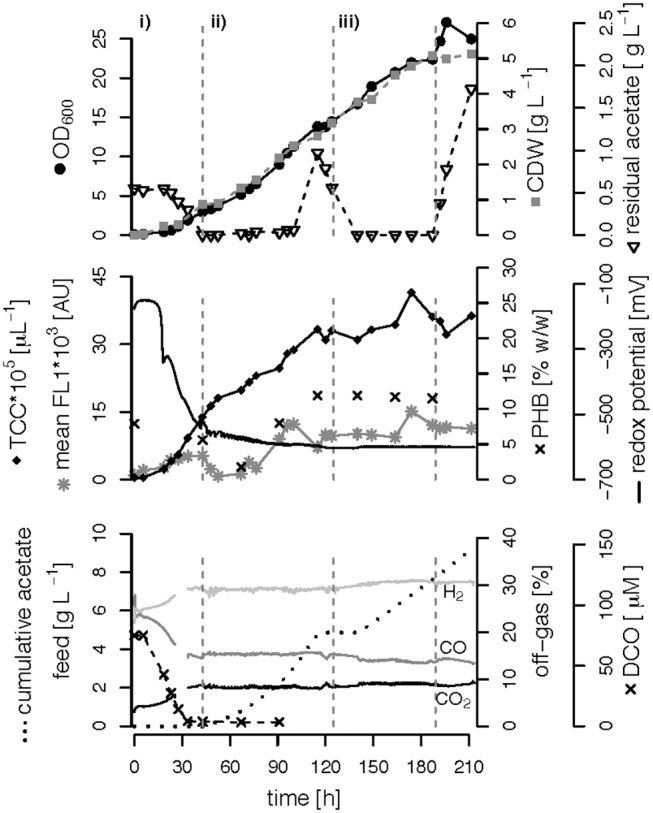
Carbon-limited fed-batch fermentation. *R. rubrum* was grown on modified RRNCO medium with continuous syngas supply. The different phases of the process are separated by gray, dashed lines: (i) batch phase during which the initial acetate is consumed; (ii) and (iii) acetate fed-batch phase. Please note that the distinction in phases (ii) and (iii) is in this experiment only for ease of clarity and coincides with the phase of a second nutrient limitation in other fed-batches. Concentrations of CO, H_2_, and CO_2_ in the off-gas are labeled in the plot. CDW: cell dry weight, TCC: total cell count, mean FL1: fluorescence signal from labeled, intracellular PHB, DCO: dissolved CO.

**Figure 4:**
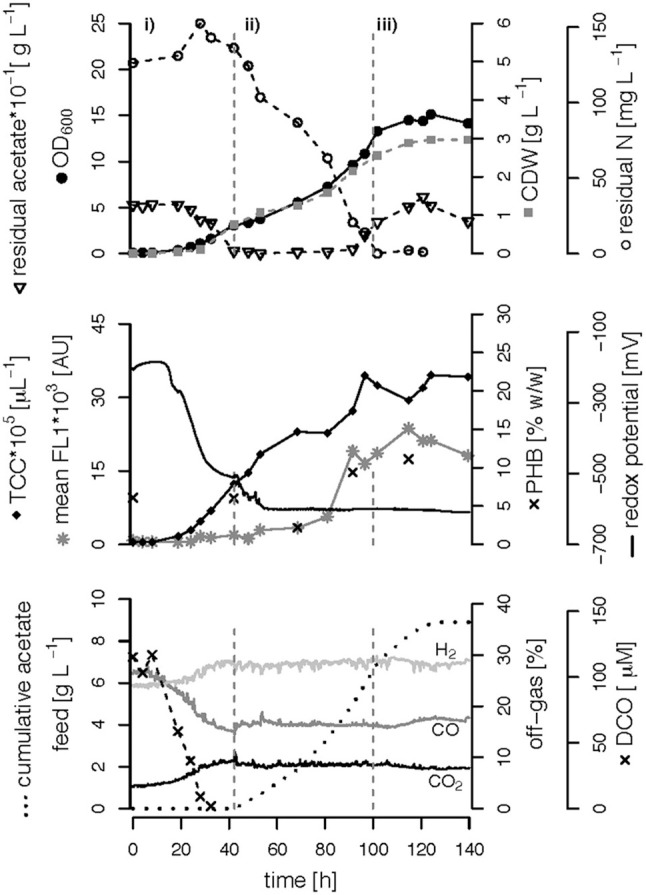
C,N-limited fed-batch fermentation. *R. rubrum* was grown on modified RRNCO medium with a reduced ammonium content and continuous syngas supply. The process is separated into three phases by gray dashed lines: (i) batch phase during which the initial acetate is consumed, (ii) acetate fed-batch, (iii) acetate fed-batch phase with C and N limitation. All abbreviations are the same as in Figure 3.

**Figure 5:**
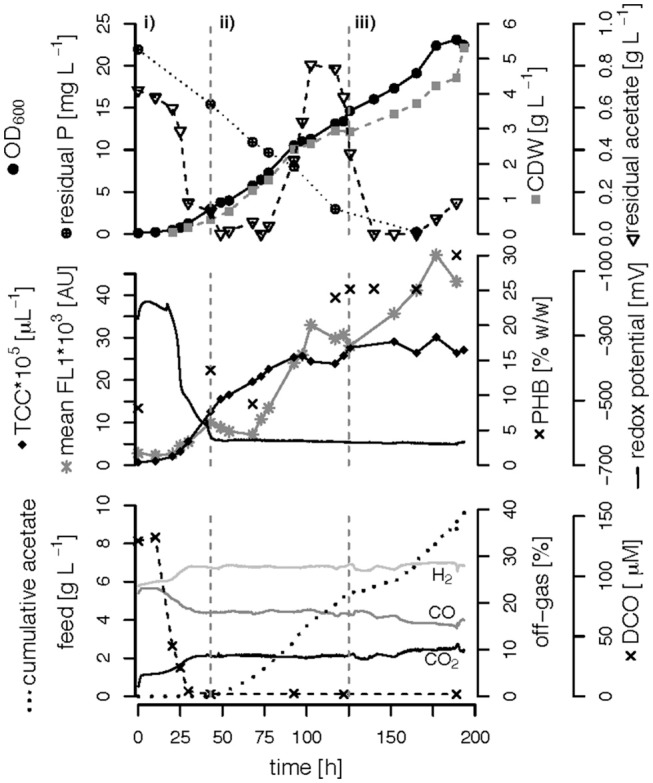
C,P-limited fed-batch fermentation. *R. rubrum* was grown on modified RRNCO medium with yeast extract as sole P source and a continuous supply of syngas. The three different growth phases of the process are separated by gray dashed lines: (i) batch phase during which the initial acetate is consumed, (ii) acetate fed-batch, (iii) acetate fed-batch C and P limitation. All abbreviations are the same as in Figure 3.

We performed three fed-batch fermentations, only C (CO and acetate)-limited (Figure 3), dual C,N-limited (Figure 4) and dual C,P-limited (Figure 5), to assess growth and in particular PHB accumulation of *R. rubrum* in more detail. During these cultivations, syngas (25% CO, 25% H_2_, 5% CO_2_, and 45% N_2_ v/v) was continuously sparged at 0.1 L L^−1^ min^−1^ into the culture broth, but the acetate supply was increased step-wise according to bacterial growth. To facilitate comparison of the fed-batch results, we defined three phases: (i) the initial batch phase during which the initial acetate was consumed along with the reduction of the redox potential, typically lasting from 0 to 43 h; (ii) the growth phase during which acetate was fed to the culture according to the biomass produced; and (iii) the PHB accumulation phase during which acetate was continued to be fed to the culture like during the growth phase, but a second nutrient (N or P, respectively) was growth-limiting. Except in case of the C-limited fed-batch, the phase (iii) corresponds to the phase (iii) of the C,P-limited fed-batch and was used to select the timeframe that was necessary for comparison of the data (Figure 3).

#### CO as Substrate During the Batch Phase in the Bioreactor vs. Shake Flasks

**Figure 6:**
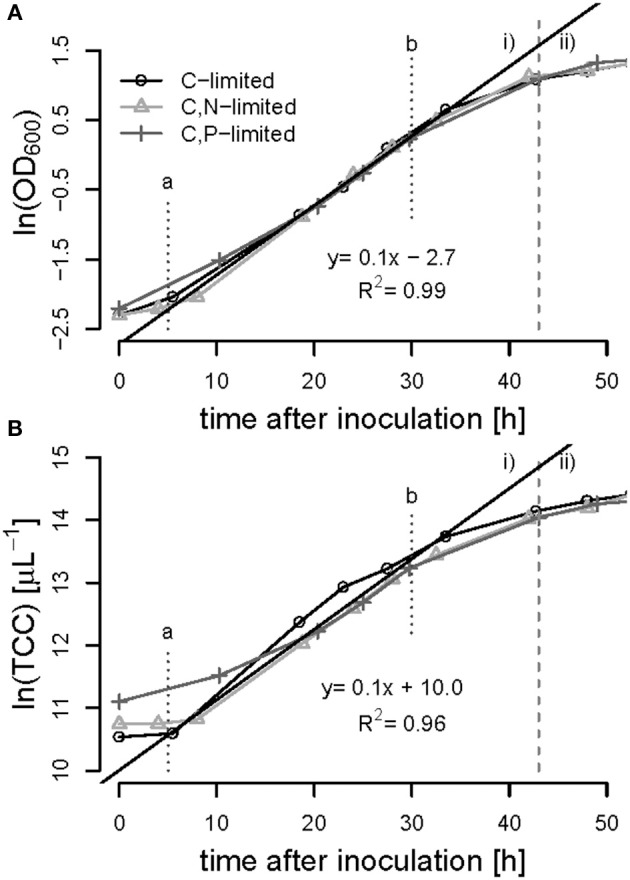
Batch-phase of *R. rubrum* on syngas and acetate in the bioreactor. The optical density [ln(OD_600_)] **(A)** and the total cell count [ln(TCC)] **(B)** was plotted against time. The gray dashed line separates the batch phase (i) from the first continuous acetate feeding phase (ii). The linear trend-line indicates the exponential growth of μ_max_ = 0.1 h^−1^ between time points a and b at 5 and 30 h of cultivation, respectively. The symbols refer to the nutrient limited phase that will follow later on.

All batch phases exhibited a lag phase of approximately 5 h during which the cultures presumably adapted from the preculture conditions (growth on fructose) to the growth on syngas and acetate. Exponential (unlimited) growth was observed between 5 and 30 h of cultivation in all bioreactor experiments showing a maximum specific growth rate of μ_max_ = 0.1 ± 0.01 h^−1^ based on OD_600_ and on TCC measurements (Figure 6). During the exponential growth phase the OD_600_ correlated with the number of cells with a conversion factor of 4.45 10^8^ cells mL^−1^ ${\text{OD}}_{600}^{-1}$ (R^2^ = 0.99), the biomass correlated with the cell number with a factor of 1.5 10^12^ cells g^−1^ biomass (*R*^2^ = 0.93).

The advantages of cultivating *R. rubrum* in a bioreactor compared to shake flasks are significant in terms of growth rates: At the end of the batch phase, typically after 43 h, we measured an OD_600_ of 3.0 ± 0.05, whereas after 48 h in shake flasks a maximal OD_600_ of 1 could be obtained (Figure 2A). In general, growth during the batch phase in the bioreactor was very reproducible for all fed-batch experiments in the bioreactor, whereas there was an error of up to 20% in shake flask cultivations (Figures 2, 6).

The DCO in the bioreactor culture broth was consumed below the detection limit (<3 μM CO) 30 h after inoculation and remained there also during phases (ii) and (iii) even though syngas was sparged continuously into the culture broth at 0.1 L L^−1^ min^−1^ (Figures 3–5). The CO-concentration in the off-gas followed the pattern of the DCO with a simultaneous increase of the H_2_ and CO_2_ concentrations in agreement with the water-gas shift reaction catalyzed by the CODH. The culture redox potential also dropped drastically from −200 mV to around −500 mV concomitantly with the complete consumption of DCO. Thereafter, it decreased approximately linearly to a value of −630 mV at the end of the batch phase or the early fed-batch phase (ii) (Figures 3–5). Consequently, the redox potential was low enough (below −380 mV), during the entire process to guarantee a full activity of the CODH, the key enzyme in the CO metabolism of *R. rubrum* (Heo et al., 2001). We could also reproduce the findings from Karmann et al. (2017) that there is no need to use reducing agents like Na_2_S to reach the low redox potential of −630 mV when working with a facultative anaerobic organism in a gas-tight bioreactor and a continuous flow of oxygen-free syngas.

#### (Multiple) Nutrient-Limited Growth During Fed-Batch Fermentation

To better compare the effect of nutrient limitations on PHB production, we consider in this section only the third phase of the fed-batch cultivations during which we had implemented (multiple) nutrient limitation (phase iii) in Figures 3–5. In case of the only C-limited fed-batch (Figure 3) the same time window was chosen as for the C,P-limited fed-batch (Figure 5). During phase (ii) the C-, C,N- and C,P-limited cultures grew to a biomasses of 3.1, 2.6, and 2.9. g L^−1^, respectively. During phase (iii) the particular growth conditions resulted in different nutrient availabilities triggering a different growth pattern and PHB productivity.

Interestingly, we found three distinct patterns for the production of biomass and PHB under (multiple) nutrient-limited growth conditions: First, in case of the fed-batch only limited in carbon (CO and acetate) the maximum PHB content in % w/w was reached quite early, already 115 h after inoculation (Figure 3). From there on the CDW continued to increase for about 100 h at a rate of almost 30 mg L^−1^ h^−1^, whereas the specific PHB productivity was 2.2 mg g^−1^ h^−1^ and the volumetric PHB productivity was at 2.9 mg L^−1^ h^−1^ during this period (Figures 5A,B). The PHB content remained constant between 11 and 12% w/w. Consequently, the PHB concentration per liter reactor volume continued to rise together with the overall increase in biomass.

**Figure 7:**
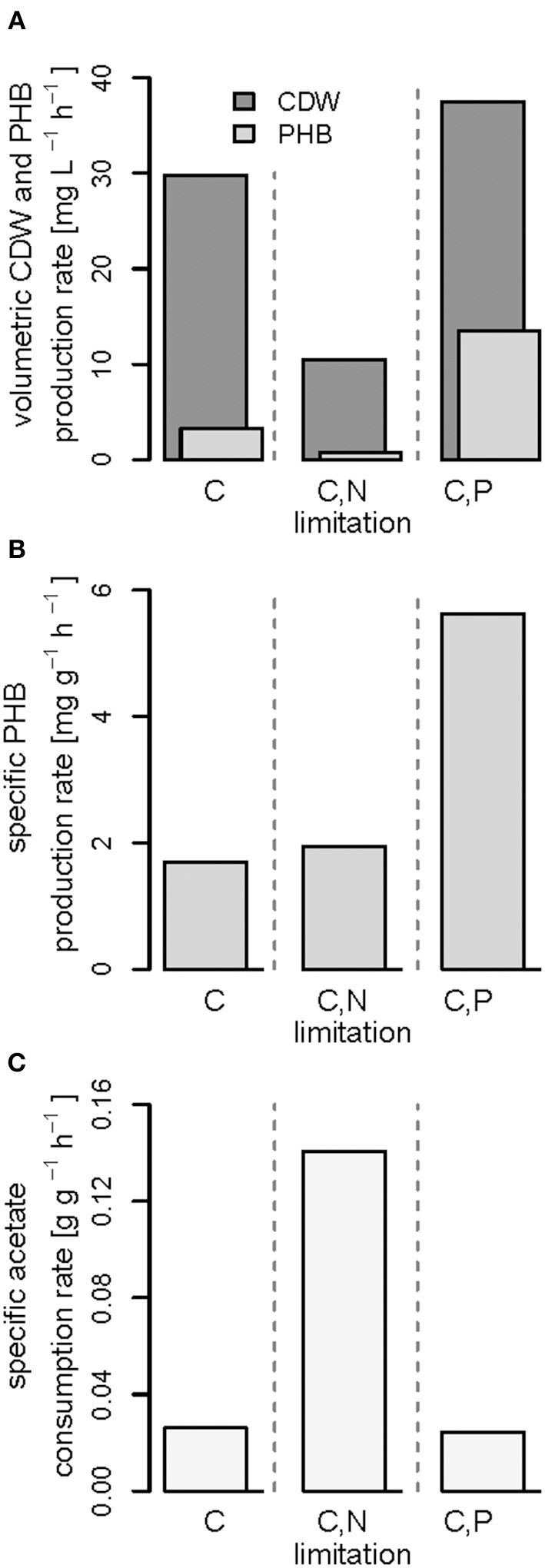
Productivities and acetate consumption rate in the (multiple) nutrient-limited growth phases. Comparison of data obtained from fed-batches with *R. rubrum* with continuous syngas and acetate feeding in the phases (iii) shown in Figures 3–5. **(A)** Volumetric productivities of cell dry weight (CDW) and PHB, **(B)** specific PHB production rate, and **(C)** specific acetate consumption rate.

Second, during C,N-limited growth (starting 100 h after inoculation), the culture exhibited a drastically reduced volumetric biomass productivity of only approximately 10 mg L^−1^ h^−1^ (Figure 7A), reducing the volumetric PHB productivity close to zero even though the specific PHB productivity of 1.95 mg g^−1^ h^−1^ was similar to the specific productivity obtained with only the C limitation (Figure 7B). Third, during C,P limitation (starting 125 h after inoculation) we measured the highest biomass productivity of 38 mg L^−1^ h^−1^ (Figure 7A) and, with 13.5 mg L^−1^ h^−1^, and 5.6 mg g^−1^ h^−1^ the highest volumetric and specific PHB production rates, respectively (Figure 7). This corresponds to a PHB productivity almost 5 times higher as during the same period in the C-limited fed-batch fermentation.

Interestingly, the intracellular PHB content in the C,P-limited culture was already two times higher compared to the C-limited fed-batch before the P limitation was fully established (at 125 h) (Figures 3, 4). It is important to mention that yeast extract, a complex nutrient compound, was the only P source in the C,P-limited culture. Hence, P limitation might already have started before a complete, measurable consumption of P since some P (in unknown form as part of yeast extract) might not have been easily accessible to *R. rubrum*. The same amount of yeast extract was present in all fed-batches, however, in the C and C,N-limited fed-batch cultivations easily available ${\text{PO}}_{4}^{3-}$ was added as principle P source.

The three different nutrient limitations also caused different specific acetate consumption rates (q_ac_) with the C,N-limited culture consuming the most acetate per g CDW and h (Figure 7C). This can be explained by the definition of q_ac_ as the ratio of the amount of acetate consumed and the low amount of biomass produced during the same time (Figures 7A,C). A similar growth and PHA accumulation pattern for N limitation has been found previously in a batch culture with *Pseudomonas putida* GPo1 and hexanoate as carbon source (Durner et al., 2001). No production of biomass or PHA was detected after depletion of the N source, even though the carbon source was still consumed. However, the authors could clearly show that the N limitation can also have the opposite effect, namely an increase in PHA productivity, when using nonanoate as carbon source for the same strain (Durner et al., 2001).

#### Influence of Feed Strategy on Final Biomass Composition

Table 1 summarizes the final biomass concentrations and compositions of the fed-batches with three different feed strategies. The highest PHB concentration, 1.6 g L^−1^, was measured at the end of the C,P-limited fed-batch. Surprisingly, the lowest PHB concentration (0.32 g L^−1^) was found under conditions of C,N limitation instead of C limitation (Table 1). The highest carbon content was measured in the biomass of the C,P-limited fed-batch which also showed the highest PHB content of 30% w/w. The reduced N-content in the C,N-limited culture medium is also reflected in the composition of the residual (PHB-free) biomass (Table 1).

**Table 1:** Concentration and composition of dried *R. rubrum* biomass at the end of the (multiple) nutrient-limited fed-batches.

	**Fed-batch**					
	**C-limited**		**C,N-limited**		**C,P-limited**	
CDW [g L^−1^]	5.1 ± 0.09		2.95 ± 0.06		5.32 ± 0.09	
PHB [g L^−1^] and [% w/w]	0.59 / 11.9		0.32 / 11.1		1.6 / 30.0	
TCC [L^−1^]	3.6 10^12^		3.4 10^12^		2.7 10^12^	
	***X***	***R***	***X***	***R***	***X***	***R***
C content [%]	48.53	47.58	49.16	48.34	51.12	49.11
H content [%]	7.24	7.27	7.06	7.07	7.32	7.46
N content [%]	5.52	6.24	5.15	5.77	6.15	8.79

*The cell dry weight (CDW) is given as mean of three independent measurements ± standard deviation. The composition of the total biomass (X) and residual (= PHB-free) biomass (R) is given as the mean value of two independent measurements that differed maximally by 1%. Values for the composition of the residual biomass were calculated after subtracting the amount of C, H, or N, respectively that was present in the form of PHB. TCC, total cell count*.

The C-limited fed-batch grew to a similar final OD_600_ and CDW as the C,P-limited one. However, the final TCC in C,P-limited culture only reached 75% of the TCC in the C-limited culture (Table 1) suggesting a difference in cell size or shape. This matches with the PHB concentration that was higher for the cells grown under C,P limitation than under C limitation. The similar TCC for a different OD_600_ of the final biomass of the C and the C,N-limited culture cannot be explained with the PHB content (Table 1). However, the cells of the C,N-limited fed-batch were hardly dividing during the entire phase (iii) which might change the cell morphology in a way that influenced the OD_600_ measurement.

#### Biomass and Product Yields for CO, Acetate, N and P

Table 2 summarizes the mass yields for C, N, and P substrates. It is striking that for all conditions tested the mass-yields for CDW and PHB on CO (Y_X/CO_ and Y_PHB/CO_) are much lower than the yields on acetate. Based on these data we hypothesize that the main portion of carbon derived from CO must have been released as CO_2_ in the off-gas. In fact, we measured a CO_2_ production in the off-gas that corresponded approximately to 60% mol/mol of the CO consumed and can therefore explain part of the low yields for CO.

**Table 2:** Total biomass and PHB yields.

	**Fed-batch**		
**Yield**	**C-limited**	**C,N-limited**	**C,P-limited**
Y_PHB/acetate_ [g g^−1^]	0.072	0.038	0.204
Y_PHB/CO_ [g g^−1^]	0.004	0.002	0.016
Y_X/acetate_ [g g^−1^]	0.628	0.353	0.681
Y_X/CO_ [g g^−1^]	0.036	0.020	0.053
Y_H2/CO_ [g g^−1^]	0.042	0.036	0.040
Y_CO2/CO_ [g g^−1^]	0.589	0.745	0.941
Y_X/N_ [g g^−1^]	17.40	17.65	n.d.
Y_X/P_ [g g^−1^]	n.d.	n.d.	112

*The yields (Y) were calculated for total biomass (X) and PHB for R. rubrum grown on syngas and acetate with the indicated nutrient limitations. The entire fed-batch process was considered, except for P and N yields, for which only the C-limited growth phase (0–100 h) was considered. n.d., not determined*.

#### Growth With Nutrient and Energy Limitations

The common growth-determining factor for all fed-batch fermentations was clearly CO. As mentioned in CO as substrate during the batch phase in the bioreactor vs. shake flasks, the DCO reached the detection limit (<3 μM) approximately 30 h after inoculation. The linear growth pattern after 30 h in fact suggests direct dependence of growth on a linearly added nutrient, which in our case can only be CO added via the gas phase. Acetate, in contrast, was fed with an increasing rate according to the growth of the culture in phases (ii) and (iii). Linear growth is a typical behavior for growth on a gaseous substrate with low solubility in water (Pirt, 1975).

Considering the low biomass and PHB yield coefficients for CO and the relatively high rate of CO_2_ production (Table 2), we argue that CO was mainly used as an energy source and only to a small extent as carbon source. This is in contradiction to the interpretation of earlier experiments with *R. rubrum* on syngas which suggested that *R. rubrum* can grow on CO as sole carbon source. It should be noted that all earlier experiments included other possible carbon sources like acetate, malate, yeast extract, or other complex compounds in the medium (Kerby et al., 1992, 1995; Do et al., 2007; Revelles et al., 2016). However, the present study is the first one applying a continuous feed of acetate. Acetate can only be assimilated by *R. rubrum* in presence of an energy source (Schultz and Weaver, 1982). In our case, only CO can take this role and is therefore most preferentially used as energy and not as carbon source.

**Figure 8:**
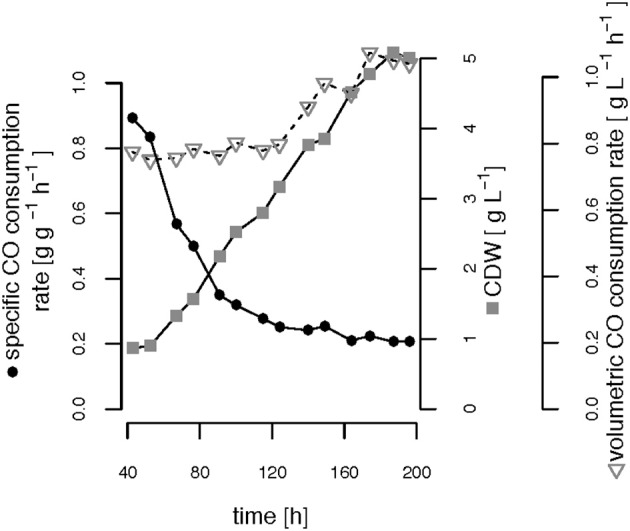
Growth-limiting CO consumption rates. The volumetric and specific CO consumption rates and the cell dry weight (CDW) are shown over time during phases (ii) and (iii) of the C-limited fed-batch.

The CO-induced energy limitation could explain why the C-limited fed-batch stopped growth at a CDW of 5.1 g L^−1^ after 189 h (Table 2, Figure 3). At this time-point acetate started to accumulate in the culture supernatant, indicating a nutrient limitation. However, no other measured nutrient suggested a limitation except CO. Due to the constant gas flow, its absolute availability does not change over time which implies that the volumetric CO consumption rate cannot increase with the total biomass in a proportional way, leading to a decreasing specific CO consumption rate (Figure 8). While in a low-biomass culture the DCO seemed to be sufficient to assimilate the available acetate, it became limiting once a critical high biomass concentration was reached. This limitation occurs at approximately 0.2 g g^−1^ h^−1^ of CO. Considering the energy of −20 kJ mol^−1^ gained from the water-gas shift reaction (Figure 1), this results in 143 J g^−1^ h^−1^, which appears to correspond to the maintenance energy required by *R. rubrum*. A measurement of the maintenance energy of *R. rubrum* and CO as energy source is, to our best knowledge, not available in literature. Interestingly, energy values were reported to be generally in the range of 80–570 J g^−1^ h^−1^ for other bacterial strains (Harder, 1997). This is in the same dimension as the empirical value from this study.

To enable unlimited or only P-limited growth also at greater cell densities, a higher availability of energy source is a necessity. A light source could be used as an additional energy source to assimilate acetate and CO-derived CO_2_ to biomass or PHB (Najafpour and Younesi, 2007). However, the scale-up of a syngas fermentation plant that does not only need additional safety measures due to CO toxicity to humans (Karmann et al., 2017) but in addition depends on energy efficient and sufficient illumination is a challenging task. Therefore, it is rather the gas-liquid mass transfer, a known challenge for fermentations involving gases that has to be improved. Previously, optimization of gas-liquid mass transfer was mainly applied to increase the availability of oxygen in culture broth (Charpentier, 1981; Arrua et al., 1990); more recently—together with the rising interest in syngas fermentations—the topic has been addressed again with the focus on CO gas-liquid mass transfer (Jones, 2007; Munasinghe and Khanal, 2010; Shen et al., 2014). New innovative fermenter types, such as the *U*-shape and/or nozzle *U*-Loop fermenter (Larsen, 2002) or a horizontally oriented rotating packed bed reactor (Shen et al., 2017) were designed to increase k_L_a coefficients. Alternatively, also high pressure bioreactors are used to increase the gas transfer (Follonier et al., 2012). An increased gas-liquid mass transfer will lead to better availability of CO as energy source and we can therefore expect higher acetate consumption rates. As a consequence, higher biomass and together with P limitation also higher PHB concentrations can be obtained.

## Conclusions

Growth and PHB production of *R. rubrum* varies greatly depending on the syngas composition used and on the availability of other nutrients. P limitation led to the highest accumulation of PHB at 30% w/w, whereas N-limited conditions inhibited growth almost completely. Furthermore, the continuous co-feeding of acetate allowed to reach higher cell densities and PHB contents than reported previously but also led to a situation of energy limitation caused by the low availability of CO which was preferentially used as energy source and only to a small extent as carbon source.

## Author Contributions

SK did the labwork and wrote the manuscript. SP and MZ supported the design of the experiments, the manuscript writing and did the proofreading.

### Conflict of Interest Statement

The authors declare that the research was conducted in the absence of any commercial or financial relationships that could be construed as a potential conflict of interest.
